# COGNITIVE-MOTOR GAMING IN OLDER ADULTS: FEASIBILITY OF TELEASSESSMENT AND TELEREHABILITATION ON FALL RISK

**DOI:** 10.1093/geroni/igac059.2605

**Published:** 2022-12-20

**Authors:** Tanvi Bhatt, Savitha Subramaniam, Spyros Kitsiou, Lakshmi Kannan, Edwina Wilson

**Affiliations:** University of Illinois at Chicago, Chicago,, Illinois, United States; University of Illinois at Chicago, Chicago,, Illinois, United States; University of Illinois at Chicago, Chicago, Illinois, United States; University of Illinois at Chicago, Chicago, Illinois, United States; University of Illinois at Chicago, Chicago, Illinois, United States

## Abstract

There is limited guidance for clinically relevant tele-assessment, and access to a comprehensive physical-activity (PA) based telerehabilitation paradigm to enhance physical function, and slow progression of frailty among older adults (OA). In this we first examined the usability, safety, and feasibility of tele-assessment and telerehabilitation program on physical, and CV function. Subsequently evaluated its compliance and effectiveness. Healthy OAs (n=23,>65 years) participated a custom-designed exergaming-based tele-exercise program (EG-BTxP) delivered in home-setting for 4 weeks (3 sessions/week) consisting of 4 exercise modules: Dancing, aerobics, cognitive-motor gaming and mind-body exercises (yoga and tai-chi) in groups of 5. Pre- and post-training, a real-time online teleassessment was performed and change in lower limb strength, endurance (30-second chair stand test), static balance (one-legged stand test, Romberg test), and dynamic balance (4-step square test), aerobic endurance (2-minute step in place test) were assessed. Participants were provided with wearable sensors and education to self-monitor heart rate during exercise and report back to health coach after each module. Participants responded positively on the qualitative usability survey and there were no adverse events . All participants were able to tolerate the teleassessment and tele-intervention with a compliance of >90%. Post-training, there were improvements in 30-second chair stand test; (p< 0.01), one-legged stand test, Romberg test, and 4-step square test, (p< 0.05). Further, number of steps in the 2-minute step-in-place test increased (p < 0.05). These findings suggest that gaming-based tele-exercise programs could be safely implemented in community-based settings to increase compliance with participation for improved physical rehabilitation outcomes.

